# Effects of cutaneous leishmaniasis on patients’ quality of life

**DOI:** 10.1186/s12879-024-09518-3

**Published:** 2024-06-18

**Authors:** Endalew Yizengaw, Endalkachew Nibret

**Affiliations:** 1 Department of Medical Laboratory Science, College of Medicine and Health Sciences, Bahir Dar University, Bahir Dar, Ethiopia; 2 Institute of Biotechnology, Bahir Dar University, Bahir Dar, Ethiopia; 3 Department of Biology, College of Science, Bahir Dar University, Bahir Dar, Ethiopia

**Keywords:** Cutaneous leishmaniasis, Quality of life, Lay gayint

## Abstract

**Introduction:**

Cutaneous Leishmaniasis (CL) is caused by protozoan parasite called *Leishmania*. It is endemic in more than 100 countries globally. Despite its vast prevalence and impact on quality of life, it is one of the most neglected tropical dermatological diseases. The CL burden has often been expressed based on the physical disfigurement caused by the disease. However, considering the impact of the disease beyond physical impairment and changes in patients’ appearance would help to better understand the disease as a public health problem. The effect of CL on patients’ quality of life was determined in this study.

**Methods:**

The data that were related to quality of life were collected using Standard one-week Dermatology Life Quality Index (DLQI) questionnaire. The questions were categorized under seven domains: symptoms & feelings, daily activities, work and school, leisure, personal relationships, and treatment. Each question was scored on a three-point scale: Very much (3), A lot (2), A little (1), Undecided (0), and Not at all (0). The sum of the scores lied between 0 and 48. A higher score shows worse quality of life. The data were entered and analysed using Statistical Package for Social Science 23. Frequencies and proportions were used to describe the data. Differences were considered statistically significant at *p* < 0.05.

**Results:**

The lives of the majority of CL patients (60.7%) were significantly affected by CL. The quality of life of patients was moderately impacted by CL in 25% of the CL patients. In 32.1% of the CL patients, the effect of CL on patients’ quality of life was very large. The quality of one CL patient’s life was extremely largely affected. The disease had a small effect on 32.1% of the CL patients. Personal relationship was the most affected domain followed by symptoms and feelings and treatment. Future study including rural regions is required.

**Conclusion:**

The Dermatology Life Quality Index demonstrates that CL has a small to extremely very large negative effect on the quality of life of patients.

## Introduction

Leishmaniasis is still one of the major public health problems worldwide especially in low- and middle-income countries [1]. It is caused by protozoan parasites of the genus *Leishmania*. It is transmitted by infected female sand fly vectors and has a digenetic life cycle [2]. The disease can be categorized into two basic clinical forms: the most severe and fatal in the absence of early treatment visceral leishmaniasis (VL) that affects internal organs and the cutaneous form that usually affects the tegumentary part [3].

Cutaneous leishmaniasis (CL) is one of the most important vector-borne diseases in Ethiopia [4, 5] and in the Lay Gayint District [6]. There are 30 million Ethiopians at risk, with an estimated 50,000 cases yearly [5, 7]. However, this is likely to be greatly underestimated not only because of the natural history of the infection but also due to the remoteness of many endemic areas and the lack of periodic surveillance in the country. Ethiopia is among the East African countries with a high burden of CL [5, 8, 9]. Like other areas, CL is characterized by three different clinical forms in Ethiopia: localized cutaneous leishmaniasis (LCL), mucocutaneous leishmaniasis (MCL), and diffuse cutaneous leishmaniasis (DCL). Besides the deformity of mucosal areas caused by the latter two forms, the disease is associated with high social stigma and discrimination in the community [10]. Since CL typically results in permanent disfiguring scars even after spontaneous healing, stigma, and poor living situations are frequently linked to it.

Cutaneous leishmaniasis is common in highland areas of the Amhara Region, northwest Ethiopia [4]. It is spreading and covering new areas that were not endemic for CL before. According to the Amhara Region Health Bureau risk estimate for 2018, there were 10 million people at risk of CL in the region. Lay Gayint is one of the CL endemic districts in the Amhara region [6]. So far, despite being an old and expanding disease, CL has not been considered a public health problem by the government and researchers in Ethiopia until recently [11]. Furthermore, data are scarce regarding the impact of CL on the quality of life of CL patients in Ethiopia generally and in the Lay Gayint District specifically. The burden of the disease is usually determined by the number of cases in terms of prevalence and/or incidence. Thus, this study aimed to determine the effects of cutaneous leishmaniasis on patients’ quality of life in Lay Gayint District, one of the most underserved areas in Ethiopia.

## Methods and materials

### Study period and design

A cross-sectional study was conducted from March 2022 to May 2022.

### Study area and setting

The study was conducted at Nefas Mewcha Hospital Leishmaniasis Treatment Centre (LTC) [6].

Nefas Mewcha Hospital is a primary hospital in Lay Gayint, northwest Ethiopia, located at 11°32’- 12°16’ N latitude to 38°12’- 38°20’ E longitude. It is 180 km by road from Bahir Dar, the capital of the Amhara National Regional State. The district of Lay Gayint has 42 kebeles. There are 9 health centres, 43 health posts, and 1 primary hospital in the district. The topography of the district is dominated by chains of mountains, hills, and valleys extending from the Tekeze Gorge (1494 m) to the Guna Mountain Summit, which has the highest elevation, 3991 m above sea level. The annual mean minimum and maximum temperatures range from 8 °C to 29 °C, respectively. The average annual rainfall of the district is 898.3 mm [12].

### Study population and data collection

Individuals with potential CL lesions presented to the LTC for diagnosis and treatment. The patients were diagnosed with CL with microscopy and clinical decisions. A slit skin smear was collected from the edge of the lesion and stained with 10% Giemsa for microscopic diagnosis. The microscopic diagnosis was confirmed by the demonstration of amastigotes in the skin smear. The classification of CL was decided based on the appearance of the lesions as described in the Guidelines for diagnosis, treatment and prevention of leishmaniasis in Ethiopia, 2013 [4]. The patients were treated with sodium stibugluconate IM (20 mg/kg/day) for 28 days following the guideline (4).

The sociodemographic and clinical information related to the disease was collected using an interviewer-administered questionnaire. The data related to quality of life was collected using the Standard one-week Dermatology Life Quality Index (DLQI) questionnaire [13]. The DLQI questionnaire measures how much a skin condition has affected the patient’s quality of life in the last seven days. Sixteen questions, customized from the DLQI were used. The questions were categorized under seven domains: symptoms & feelings, daily activities, work and school, leisure, personal relationships, and treatment. Each question, the extent to which the quality of CL patient was affected, was scored on a three-point scale: Very much (3), A lot (2), A little (1), Undecided (0), and Not at all (0). The sum of the scores lay between 0 and 48. A higher score shows a worse quality of life. According to the scores obtained on DLQI, the effect of disease on quality of life was interpreted as follows: no effect on patient’s life (DLQI score 0–2), small effect (3–9), moderate effect (10–16), very large effect (17–32), and extremely large effect (33–48) [13]. Significant effects comprised moderate, large, and extremely large effects.

### Statistical analysis

The data were entered and analyzed using Statistical Package for Social Science 23 (SPSS-23). Frequencies and proportions were used for the descriptive analysis of the data. Unless otherwise indicated, results were expressed as median with the interquartile range. Differences were considered statistically significant at *p* < 0.05. A GraphPad prism (Prism 9) was used to plot the age and sex distribution of CL patients. Mann-Whitney test was used to assess statistical differences between the ages of females and males. Differences were considered statistically significant at *p* < 0.05.

## Results

### Socio-demographic characteristics

**Fig. 1:**
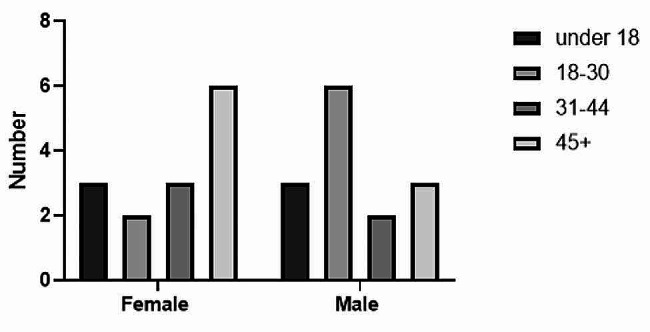
Age and sex distribution of cutaneous leishmaniasis patients at Nefas Mewcha Hospital, 2022

A total of 28 CL patients were included in this study. We recruited an equal number of female and male CL patients, each was 14 (50.0%) (Fig. 1). The minimum and maximum ages were 7 and 78 years old, respectively. The patients’ median age with an interquartile range was 32.50 [18.5–49.5] years. The median age with an interquartile range of the females and males was 36 [22.75–50] and 24 [16.75–41] years, respectively. There was no significant difference in age between female and male patients (36 [22.75–50] and 24 [16.75–41], *p* = 0.3946). Half of the patients were in the age group of 7–30 years old (Fig. 1). All the patients were from CL endemic rural kebeles of Lay Gayint District.

### Clinical profiles

Twenty (71.4%) and eight (28.6%) of the patients were positive by microscopic and clinical examination, respectively (Table 1). The great majority (92.9%) of the patients were primary patients, diagnosed with CL for the first time and only two patients were repeated patients, diagnosed with CL for the second time. Ten (35.7%) of the patients had six months and below a duration of illness at the time of recruitment and 13 (46.4%) of the patients had a duration of illness between 7 and 12 months. Three (10.7%) and two (7.1%) of the patients had a duration of illness between 13 and 24 months and above 24 months, respectively (Table 1).

**Table 1:** Clinical profiles of CL patients at Nefas Mewcha Hospital, 2022

Variables	Category	Number	%
Microscopy	Positive	20	71.4
	Negative	8	28.6
	Total	28	100.0
Diagnosis	Primary	26	92.9
	Repeat	2	7.1
	Total	28	100.0
Previous Treatment	Yes	15	53.6
	No	13	46.4
	Total	28	100.0
Type of Treatment	Traditional	13	86.7
	Anti-leishmanial	2	13.3
	Total	15	100.0
CL Type	LCL	11	39.3
	DCL	9	32.1
	MCL	8	28.6
	Total	28	100.0
Pain	Yes	15	53.6
	No	13	46.4
	Total	28	100.0
Itching	Yes	24	85.7
	No	4	14.3
	Total	28	100
Discharge (Purulent secretion)	Yes	12	42.9
	No	16	57.1
	Total	28	100.0
Bad smell	Yes	10	35.7
	No	18	64.3
	Total	28	100.0
Impairment	Yes	4	14.3
	No	24	85.7
	Total	28	100.0

The majority (53.6%) of the patients had previous treatment history (Table 1). Thirteen (86.7%) of the patients with a history of previous treatment were treated with traditional drugs and only two of the CL patients were treated with anti-leishmanial drugs of a standard regimen (Table 1). Eleven (39.3%) of the patients were presented with LCL clinical form, a disease characterized by single or multiple localized lesions on exposed areas of skin. Nine (32.1%) were of DCL type, characterized by numerous non-ulcerating nodular lesions distributed in different body parts. Andeight (28.6%) were of the MCL clinical forms, affecting the mucosa of the nose and mouth that is characterized by inflammatory response responsible for the disfiguring of affected tissue (Table 1).

The majority, 15 (53.6%) of the CL patients responded that they had symptoms of pain. The great majority, 24 (87.5%) of the CL patients had a history of itching (Table 1). Twelve (42.9%) of the CL patients had discharge from the lesion and ten of these had a lesion with a bad smell (Table 1). Four (14.3%) of the CL patients had impairment of one and/or more of their body parts (Table 1).

### Effect of cutaneous leishmaniasis on the patient’s quality of life

The lives of the majority of CL patients (60.7%) were significantly affected by CL. The quality of life of patients was moderately impacted by CL in 25% (7/28) of the CL patients. In 32.1% (9/28) of the CL patients, the effect of CL on patients’ quality of life was very large. The quality of one CL patient’s life was extremely largely affected by the disease. The diseases had a small effect on 32.1% (9/28) of the CL patients and did not affect the lives of only two CL patients (Table 2).

**Table 2:** Quality of life measures

Variables	Number	%
No effect	2	7.1
Small effect	9	32.1
Moderate effect	7	25.0
Very large effect	9	32.1
Extremely large effect	1	3.6
Total	28	100.0

## Discussion

In this study, we have studied the effect of CL in the quality of life of selected cutaneous leishmaniasis cases from Lay Gayint district. The majority of patients with cutaneous leishmaniasis are not coming to health facilities for diagnosis and treatment early in Ethiopia including the study area. They are rather using traditional medicine and religious practices. This is because of low awareness about the disease and lack of diagnosis and treatment centers. This again results in high reduction in the quality of life of patients as there is high social stigma and discrimination. This shows the need for rapid diagnosis and appropriate treatment in the health centers.

The physical deformity brought on by cutaneous leishmaniasis has frequently been utilized to determine the disease burden on the community and/or individuals, and it is stated in terms of prevalence and/or incidence. Nevertheless, expanding our view to consider the disease’s repercussions that go beyond physical impairment and changes in patient’s appearance would help us understand the disease as a public health problem. This will enable a better understanding of the impact of the disease and the rational allocation of public resources. In this study, the effect of CL on patients’ quality of life was assessed. This is the first study to assess the impact of cutaneous leishmaniasis on the patient’s quality of life using the Standard one-week Dermatology Life Quality Index in Ethiopia. The disease is significantly affecting the quality of life of patients with CL. The quality of life of the majority of study participants, 92.8% (26/28) was affected by cutaneous leishmaniasis. The degree of the CL impact on CL patients’ quality of life ranged from small effect (32.1%) to extremely large effect (3.6%). The majority (57.1%) of the CL patients’ quality of life was affected moderately and very largely.

The degree of the impact of the disease on the quality of life of patients is associated with the severity of the disease. Similar results have been indicated from different studies [14]. The disease significantly affects 81.8%, 75%, and 88.9% of the quality of life of patients with LCL, MCL, and DCL clinical forms respectively. A similar result has been reported from Iran [15] where they showed that the type of LCL lesion had a significant effect on the patient’s quality of life. The DLQI score in patients with papular LCL lesions was better than in those with nodular and plaque lesions. The impact of CL on the quality of life of patients with diffused cutaneous leishmaniasis ranges from small to extremely large. The effect of CL on the quality of life of half of the CL patients with mucocutaneous leishmaniasis was very large (Table 3).

**Table 3:** The impact of CL on the quality of life with clinical forms

Quality of life measures	Clinical form			
	LCL	DCL	MCL	Total
No effect	2	0	0	2
Small effect	6	1	2	9
Moderate effect	2	3	2	7
Very large effect	1	4	4	9
Extremely large effect	0	1	0	1
Total	11	9	8	28

The domain related to personal relationships was the most affected domain followed by symptoms and feelings, treatment and leisure. Patients with CL lesions might develop a broad spectrum of psychosocial sufferings. They usually get anxious, shy, ashamed, stressed, and depressed. They develop feelings of embarrassment, sadness, suicidal thoughts, and a decrease in self-confidence, self-esteem, self-contempt, and self-awareness, leading to a low quality of life. These lead patients to perceive or interpret negatively some direct or indirect reactions from partners, relatives, and the general population. Patients with CL lesions might also develop the concept of loss of beauty because of the lesions and the scars [16, 17]. Moreover, the impact of unwanted changes in body image is considered bad luck that will negatively affect their social interaction and diminish the marriage opportunities of the person suffering from CL lesion and/or scar. A similar report has revealed that patients with CL lesions reported feelings of inferiority and the idea that the disease is equal to an apparent social disadvantage [16]. It has been shown that LCL patients with active lesions who were taking standard treatment plus psychotherapy showed a more pronounced improvement in the quality of life at the end of the study after 8 weeks than those who were taking the standard LCL treatment only [18]. The CL patients might also feel their chances to find employment, studies, or marriage jeopardized. This will again result in psychological and emotional consequences [14].

## Conclusions

This study shows that cutaneous und mucosal leishmaniasis may reduce the quality of life significantly mainly after long duration of the disease. The Dermatology Life Quality Index demonstrates that CL has a small to extremely very large negative effect on the quality of life of patients with cutaneous leishmaniasis. Studies with larger numbers of patients including rural regions are needed to evaluate the effects of location, number, size, and duration of CL lesions, as well as the effects of treatment and residual scars, on the quality of life of patients.

## Data Availability

All data generated or analyzed during this study are included in this published article.

## Abbreviations

APHI: Amhara Public Health Institute
CL: Cutaneous Leishmaniasis
DCL: Diffuse Cutaneous Leishmaniasis
DLQI: Dermatology Life Quality Index
LCL: Localised Cutaneous Leishmaniasis
LTC: Leishmaniasis Treatment Centre
MCL: Mucocutaneous Leishmaniasis
RECCS: Research and Ethical Review Committee of the College of Science
VL: Visceral Leishmaniasis
